# Inflammatory mechanisms in ischemic stroke: therapeutic approaches

**DOI:** 10.1186/1479-5876-7-97

**Published:** 2009-11-17

**Authors:** Shaheen E Lakhan, Annette Kirchgessner, Magdalena Hofer

**Affiliations:** 1 Global Neuroscience Initiative Foundation, Los Angeles, CA, USA

## Abstract

Acute ischemic stroke is the third leading cause of death in industrialized countries and the most frequent cause of permanent disability in adults worldwide. Despite advances in the understanding of the pathophysiology of cerebral ischemia, therapeutic options remain limited. Only recombinant tissue-plasminogen activator (rt-PA) for thrombolysis is currently approved for use in the treatment of this devastating disease. However, its use is limited by its short therapeutic window (three hours), complications derived essentially from the risk of hemorrhage, and the potential damage from reperfusion/ischemic injury. Two important pathophysiological mechanisms involved during ischemic stroke are oxidative stress and inflammation. Brain tissue is not well equipped with antioxidant defenses, so reactive oxygen species and other free radicals/oxidants, released by inflammatory cells, threaten tissue viability in the vicinity of the ischemic core. This review will discuss the molecular aspects of oxidative stress and inflammation in ischemic stroke and potential therapeutic strategies that target neuroinflammation and the innate immune system. Currently, little is known about endogenous counterregulatory immune mechanisms. However, recent studies showing that regulatory T cells are major cerebroprotective immunomodulators after stroke suggest that targeting the endogenous adaptive immune response may offer novel promising neuroprotectant therapies.

## Introduction

Stroke is the third leading cause of death in industrialized countries [1] and the most frequent cause of permanent disability in adults worldwide [2]. Three months following a stroke, 15-30% of stroke survivors are permanently disabled and 20% require institutional care [3]. Deficits can include partial paralysis, difficulties with memory, thinking, language, and movements. In the Western world, over 70% of individuals experiencing a stroke are over 65 years of age. Since life expectancy continues to grow, the absolute number of individuals with stroke will further increase in the future.

The most common cause of stroke is the sudden occlusion of a blood vessel by a thrombus or embolism, resulting in an almost immediate loss of oxygen and glucose to the cerebral tissue. Although different mechanisms are involved in the pathogenesis of stroke, increasing evidence shows that ischemic injury and inflammation account for its pathogenic progression [4]. Cerebral ischemia triggers the pathological pathways of the ischemic cascade and ultimately causes irreversible neuronal injury in the ischemic core within minutes of the onset [5].

However, a much larger volume of brain tissue surrounding this ischemic core, known as the penumbra, can be salvaged if cerebral blood flow is promptly restored. Thus, the original definition of the ischemic penumbra referred to areas of brain that were damaged but not yet dead, offering the promise that if proper therapies could be found, one could rescue brain tissue after stroke and reduce post-stroke disability.

Despite advances in the understanding of the pathophysiology of cerebral ischemia, therapeutic options for acute ischemic stroke remain very limited [2]. Only one drug is approved for clinical use for the thrombolytic treatment of acute ischemic stroke in the US and that is intravenous recombinant tissue plasminogen activator (rt-PA). When delivered within three hours after symptom onset, rt-PA reduces neurological deficits and improves the functional outcome of stroke patients. However, this improvement in recovery is achieved at the expense of an increased incidence in symptomatic intracranial hemorrhage, which occurs in ~6% of patients. Furthermore, since the large majority of patients with acute ischemic stroke do not go to the hospital within three hours of stroke onset most do not receive rt-PA treatment [6]. Consequently, the successful treatment of acute ischemic stroke remains one of the major challenges in clinical medicine.

This review will provide a brief overview of the current understanding of the inflammatory mechanisms involved in an acute ischemic stroke and the neuroprotective agents that can curtail neuroinflammation and potentially show utility in the treatment of stroke. Neuroprotective treatments are therapies that block the cellular, biochemical, and metabolic elaboration of injury during exposure to ischemia. Of the more than 100 neuroprotective agents that reached randomized clinical trials in focal ischemic stroke, none has proven unequivocally efficacious, despite success seen in preceding animal studies [7]. However, the failed trials of the past have greatly increased our understanding of the fundamental biology of ischemic brain injury and have laid a strong foundation for future advance. New anti-inflammatory targets continue to be identified, which is an important area for translational medicine in acute stroke. Overall, the prospects for safe neuroprotective therapies to improve stroke outcome remain promising [8]

### Ischemic cascade

Acute ischemic stroke accounts for about 85% of all cases while hemorrhagic stroke is responsible for almost 15% of all strokes. Ischemic stroke results from the sudden decrease or loss of blood circulation to an area of the brain, resulting in a corresponding loss of neurological function. It is a nonspecific term encompassing a heterogeneous group of etiologies including thrombosis, embolism, and relative hypoperfusion. In most cases, the cause is atherothrombosis of large cervical or intracranial arteries, or embolism from the heart.

**Figure 1:**
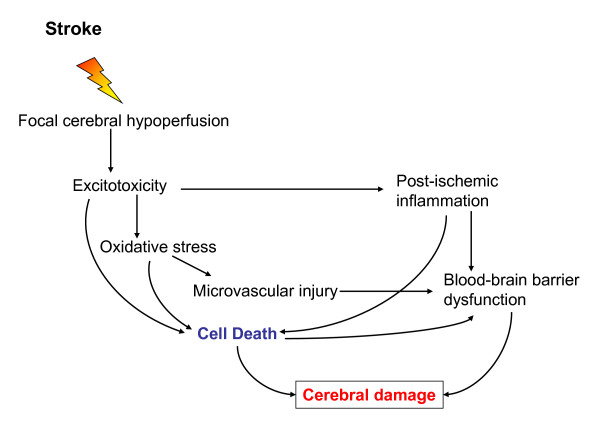
**Ischemic cascade leading to cerebral damage**. Ischemic stroke leads to hypoperfusion of a brain area that initiates a complex series of events. Excitotoxicity, oxidative stress, microvascular injury, blood-brain barrier dysfunction and postischemic inflammation lead ultimately to cell death of neurons, glia and endothelial cells. The degree and duration of ischemia determines the extent of cerebral damage.

Within seconds to minutes after the loss of blood flow to a region of the brain, the ischemic cascade is rapidly initiated, which comprises a series of subsequent biochemical events that eventually lead to disintegration of cell membranes and neuronal death at the center/core of the infarction. Ischemic stroke begins with severe focal hypoperfusion, that leads to excitotoxicity and oxidative damage which in turn cause microvascular injury, blood-brain barrier dysfunction and initiate post-ischemic inflammation. These events all exacerbate the initial injury and can lead to permanent cerebral damage (see Figure 1). The amount of permanent damage depends on several factors: the degree and the duration of ischemia and the capability of the brain to recover and repair itself [5].

As a result of residual perfusion from the collateral blood vessels, regions where blood flow drops to approximately 30 ml/100 g/min ischemic cascade progresses at a slower rate. Neuronal cells may tolerate this level of reduced (20-40% of control values) blood flow for several hours from the stroke onset with full recovery of function following restoration of blood flow [9].

In the center of the ischemic region cells undergo anoxic depolarization and they never repolarize. While in the penumbral region, the cells can repolarize at the expense of further energy consumption and depolarize again in response to elevated levels of extracellular glutamate and potassium ions. Such repetitive depolarizations called "peri-infarct depolarizations" lead to the increased release of the excitatory neurotransmitter glutamate with resulting excitotoxic cell damage [10]. Ultimately, the severity of functional and structural changes in the brain caused by ischemia will depend on its degree and duration.

Hyperbaric (HBO) and normobaric oxygen (NBO) therapies attempt to increase the partial pressure of oxygen to the tissue and thereby limit the damage caused by hypoperfusion. However, three clinical trials of hyperbaric oxygen therapy failed to show efficacy [11]. Normobaric, high-flow oxygen therapy was shown to cause a transient improvement of clinical deficits and MRI abnormalities in a sub-group of patients with acute ischemic stroke. Further studies are needed to investigate the safety and efficacy of hyperoxia as a stroke therapy [12].

### Oxidative stress

Oxidative stress contributes to the pathogenesis of a number of neurological conditions including stroke. Oxidative stress is defined as the condition occurring when the physiological balance between oxidants and antioxidants is disrupted in favor of the former with potential damage for the organism. Oxidative stress leading to ischemic cell death involves the formation of ROS/reactive nitrogen species through multiple injury mechanisms, such as mitochondrial inhibition, Ca^2+ ^overload, reperfusion injury, and inflammation [13]. Plenty of ROS are generated during an acute ischemic stroke and there is considerable evidence that oxidative stress is an important mediator of tissue injury in acute ischemic stroke [14]. Brain ischemia generates superoxide (O_2_^-^), which is the primary radical from which hydrogen peroxide is formed. Hydrogen peroxide is the source of hydroxyl radical (OH). Nitric oxide is a water- and lipid-soluble free radical that is produced from L-arginine by three types of nitric oxide synthases (NOS). Ischemia causes an increase in NOS type I and III activity in neurons and vascular endothelium, respectively. At a later stage, elevated NOS type II (iNOS) activity occurs in a range of cells including glia and infiltrating neutrophils. Thus, free radicals are regarded as an important therapeutic target for improving the outcome of an ischemic stroke. Several compounds with significant antioxidant properties including ebselen [15], and resveratrol [16], a natural phytoalexin found in some dietary sources such as grapes and red wine, have been demonstrated to reduce stroke-related brain damage in animal models.

### The transcription factor Nrf2

Nuclear factor erythroid-related factor 2 (Nrf2) is a transcription factor that regulates an expansive set of antioxidant genes that act in synergy to remove ROS through sequential enzymatic reactions [17].

**Figure 2:**
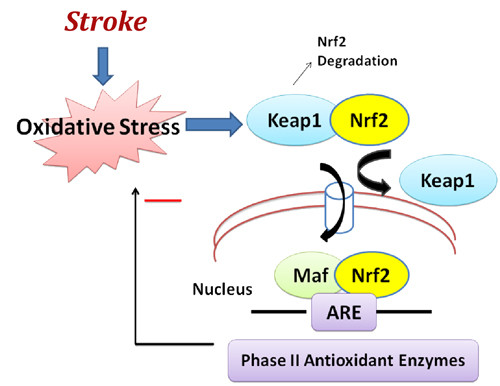
**Nuclear erythroid-related factor 2 (Nrf2) anti-oxidant signaling in acute ischemic stroke**. Nrf2 is the principal transcription factor that regulates antioxidant response element (ARE)-mediated expression of phase II detoxifying antioxidant enzymes. Under normal conditions, Nrf2 is sequestered in the cytoplasm by an actin-binding (Kelch-like) protein (Keap1); on exposure of cells to oxidative stress, Nrf2 dissociates from Keap1, translocates into the nucleus, binds to ARE, and transactivates phase II detoxifying and antioxidant genes. Among the spectrum of antioxidant genes controlled by Nrf2 are catalase, superoxide dismutase (SOD), glutathione reductase, and glutathione peroxidase.

Nrf2 gene targets, collectively referred to as phase II genes, are involved in free radical scavenging, detoxification of xenobiotics, and maintenance of redox potential. Nrf2 is normally localized to the cytoplasm, tethered to the regulatory protein, kelch-like erythroid cell-derived protein with CNC homology associated protein 1 (Keap1) (Figure 2). Oxidative stress, or electrophilic agents that mimic oxidative stress, can modify key sulfhydryl group interactions in the Keap-Nrf2 complex, allowing dissociation and nuclear translocation of Nrf2. When activated, Nrf2 specifically targets genes bearing an antioxidant response element (ARE) within their promoters such as heme oxygenase 1, 1-ferritin, and glutathione peroxidase, which maintain redox homeostasis and influence the inflammatory response. Wide ranges of natural and synthetic small molecules are potent inducers of Nrf2 activity. These molecules have been identified from diverse chemical backgrounds including isothiocyanates, which are abundant in cruciferous vegetables, heavy metals, and hydroperoxides.

Several studies have shown that increasing Nrf2 activity is highly neuroprotective in *in vitro *models that stimulate components of stroke damage, such as oxidative glutamate toxicity, H_2_O_2 _exposure, and Ca^2+ ^overload [18]. Administration of the well characterized Nrf2 inducer, *tert*-butylhydroquinone (tBHQ), a metabolite of the widely used food antioxidant butylated hydroxyanisole, significantly improved sensorimotor and histological outcome in two models of I/R in rats and mice [19]. Within this injury paradigm, Nrf2 activation before stroke was able to salvage the cortical penumbra but not the stroke core. Clear differences in stroke outcome were found as early as 24 hours after reperfusion. Moreover, prophylactic treatment improved functional recovery up to one month after transient MCAO suggesting that previous Nrf2 activation may reduce neuronal cell death during delayed apoptosis and inflammation long after stroke onset.

Conversely, Nrf2-deficient mice are significantly more prone to ischemic brain injury and neurological deficits than WT mice. Deletion of the Nrf2 gene renders animals more susceptible to various stressors mainly because of the failure to induce phase II enzymes. Furthermore, an Nrf2 inducer was able to reverse neuronal cell death induced by the free radical donor *tert*-butylhydroperoxide (*t*-BuOOH) [19]. The MCAO and reperfusion model is known to induce a transient focal ischemic cascade that uniquely includes a substantial surge of free radical damage.

### Ischemia/reperfusion (I/R) injury

The ischemic cascade usually goes on for hours but can last for days, even after restoration of blood circulation. Although reperfusion of ischemic brain tissue is critical for restoring normal function, it can paradoxically result in secondary damage, called ischemia/reperfusion (I/R) injury.

The definitive pathophysiology regarding I/R injury still remains obscure; however, oxidative stress mediators such as reactive oxygen species (ROS) released by inflammatory cells around the I/R injured areas are suggested to play a critical role [20]. The increase in oxygen free radicals triggers the expression of a number of pro-inflammatory genes by inducing the synthesis of transcription factors, including NF-κB, hypoxia inducible factor 1, interferon regulator factor 1 and STAT3. As a result, cytokines are upregulated in the cerebral tissue and consequently, the expression of adhesion molecules on the endothelial cell surface is induced, including intercellular adhesion molecule 1 (ICAM-1), P-selectin and E-selectin which mediate adhesion of leukocytes to endothelia in the periphery of the infarct [21].

Furthermore, the complement cascade has been shown to play a critical role in I/R injury [22]. In addition to direct cell damage, regional brain I/R induces an inflammatory response involving complement activation and generation of active fragments such as C3a and C5a anaphylatoxins. Expression of C3a and complement 5a receptors was found to be significantly increased after middle cerebral artery occlusion (MCAO) in the mouse indicating an active role of the complement system in cerebral ischemic injury. Complement inhibition resulted in neuroprotection in animal models of stroke [23].

### Post-ischemic inflammation

Although for many years the CNS was considered an immune-privileged organ, it is now well accepted that the immune and the nervous system are engaged in bi-directional crosstalk. Moreover, mounting data suggest that in the brain, as in peripheral organs, inflammatory cells participate in tissue remodeling after injury.

Microglial cells are the resident macrophages of the brain and play a critical role as resident immunocompetent and phagocytic cells in the CNS. Ekdahl and colleagues [24] reported an increased number of activated microglial cells up to 16 weeks after two hour MCAO in rats. After activation by ischemia, microglia can transform into phagocytes and they can release a variety of substances many of which are cytotoxic and/or cytoprotective. Microglia may exert neuroprotection by producing neurotrophic molecules such as brain-derived neurotrophic factor (BDNF), insulin-like growth factor I (IGF-I), and several other growth factors. There is substantial evidence that activated microglial cells in response to ischemia have the potential of releasing several pro-inflammatory cytokines such as TNF-α, IL-1β, and IL-6, as well as other potential cytotoxic molecules including NO, ROS, and prostanoids [25].

Astrocytes, like microglia, are capable of secreting inflammatory factors such as cytokines, chemokines, and NO [26]. Cytokines upregulate the expression of cell adhesion molecules (CAMs). Within four to six hours after ischemia onset, circulating leukocytes adhere to vessel walls and migrate into the brain with subsequent release of additional pro-inflammatory mediators and secondary injury in the penumbra. Neutrophils are the earliest leukocyte subtype to show substantial upregulation in gene expression studies and to infiltrate areas of brain ischemia (see below). Recently, Shichita et al. [27] demonstrated an infiltration of γdT cells 3 days after the onset of ischemia in a mouse model, along with a production of IL-17 which amplify the inflammatory cascade. IL-23 from infiltrating macrophages appear to produce Il-23 which attracts the infiltrating γdT cells. Blocking a specific γdT cell receptor with an antibody effectively reduced three-day infarct volumes, even when treatment was initiated at 24 hours after onset of cerebral ischemia. Targeting these γdT cells may offer a clinical opportunity with a longer therapeutic window to prevent the secondary inflammatory expansion of cerebral damage after stroke.

**Figure 3:**
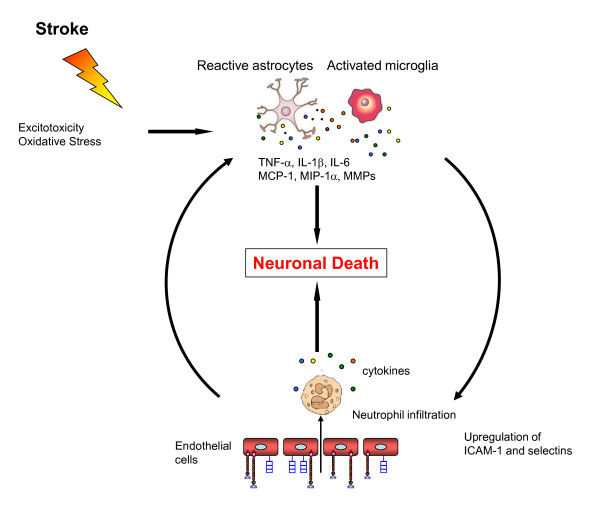
**Postischemic inflammatory response**. Excitotoxicity and oxidative stress caused by the initial ischemic event activate microglia and astrocytes which react by secreting cytokines, chemokines and matrix metalloproteases (MMP). These inflammatory mediators lead to an upregulation of cell adhesion molecules on endothelial cells, allowing blood-derived inflammatory cells, mainly neutrophils, to infiltrate the ischemic brain area. Neutrophils themselves also secrete cytokines which cause a further activation of glial cells. These processes all result in neuronal cell death and enhance the damage to the ischemic brain.

The described post-ischemic neuroinflammatory changes lead to dysfunction of the blood-brain barrier, cerebral edema, and neuronal cell death (summarized in Figure 3). Therefore, therapeutic targeting of the neuroinflammatory pathways in acute ischemic stroke has become an important area of research in translational medicine.

### Cytokines and brain inflammation

Cytokines are a group of small glycoproteins that are produced in response to an antigen and were originally described as mediators for regulating the innate and adaptive immune systems. Cytokines are thus upregulated in the brain in a variety of diseases, including stroke. In the brain, cytokines are expressed not only in the cells of the immune system, but are also produced by resident brain cells, including neurons and glia [28]. In addition, it has been shown that peripherally derived cytokines are involved in brain inflammation. Thus, peripherally derived mononuclear phagocytes, T lymphocytes, NK cells and polymorphonuclear leukocytes produce and secrete cytokines and might contribute to inflammation of the CNS [29].

The most studied cytokines related to inflammation in acute ischemic stroke are tumor necrosis factor-α (TNF-α), the interleukins (IL), IL-1β, IL-6, IL-20, IL-10 and transforming growth factor (TGF)-β. While IL-1β and TNF-α, appear to exacerbate cerebral injury, TGF-β and IL-10 may be neuroprotective [30,31]. Increased production of pro-inflammatory cytokines and lower levels of the anti-inflammatory IL-10 are related to larger infarctions and poorer clinical outcome.

Elevated IL-1β mRNA expression occurs within the first 15-30 min after permanent MCAO and elevated IL-1β protein expression occurs a few hours later and remains elevated for up to 4 days [32]. There are studies that correlate an increase in the levels of IL-1β after ischemia with worsening of the infarct severity. For example, Yamasaki et al [33] demonstrated that intraventricular injection of recombinant IL-1β after MCAO increases the formation of brain edema, the volume of the size and the influx of neutrophils. In addition, IL-1β deficient mice presented smaller infarcts in comparison with wild-type mice [34].

High circulating IL-1β elevates circulating IL-6, another well known cytokine that is upregulated following cerebral ischemia [35]. Moreover, the serum level of IL-6 correlates with brain infarct volume [36] and is a powerful predictor of early neurological deterioration [37]. On the other hand, Clark et al [38] demonstrated that infarct size and neurological function were not different in animals deficient in IL-6 after transient CNS ischemia. This suggests that IL-6 does not have a direct influence on acute ischemic injury.

IL-20 is induced when IL-1β modulates p38 MAPK and the NF-κB pathway. IL-20 in turn induces the production of IL-6. Inhibition of IL-20 by a specific mAb significantly ameliorated the brain ischemic infarction in rats following MCAO [39].

Several approaches are under investigation for managing IL-1 in stroke (Table 1). IL-1 acts via membrane receptors (IL-1R), which can be blocked by a receptor antagonist (IL-1RA). In a randomized trial for acute stroke, IL-1RA readily crossed the blood-brain barrier, was safe to use, and seemed to afford some benefit, particularly for patients with cortical infarcts [40].

**Table 1:** Clinical studies of agents targeting inflammatory pathways in acute ischemic stroke.

Neuroprotective Agent	Mode of Action	Reference
Recombinant human IL-1RA	Interleukin-1 receptor antagonist	[67]

Enlimomab	Anti-ICAM-1 monoclonal antibody	[68]

Tirilazad	Lipid peroxidation inhibitor	[69]

UK-279, 276	Neutrophil inhibitory factor	[70]

Cerovive (NXY-059)	Nitrone-based free radical trapping agent	[71,72]

Acetaminophen (Paracetamol)	Anti-pyretic effect	[73]

Minocycline	Anti-inflammatory	[74]

Ginsenoside	Ca^2+ ^channel antagonist	[75]

Edaravone MCI-186	Free radical scavenger	[76]

ONO-2506 (Arundic Acid)	Astrocyte modulator	[77]

Adapted from Shah et al., 2009 [78].

IL-10 is an anti-inflammatory cytokine that acts by inhibiting IL-1 and TNF-α, and by suppressing cytokine receptor expression and receptor activation as well. As a consequence, IL-10 could provide neuroprotection in acute ischemic stroke. Both central and systemic administration of IL-10 to rats subjected to MCAO significantly reduced infarct size 30 min to three hours post MCAO [30]. In acute ischemic stroke, elevated concentrations of IL-10 in CSF have been found [41]. Moreover, patients with low plasma levels (<6 pg/ml) of IL-10 during the first hours after stroke were three times more likely to have worsening neurological symptoms within 48 hours following the stroke [37]. IL-10 also seems to mediate the reduction in infarct size by regulatory T cells (see below).

### Chemokines and brain inflammation

Chemokines, for example, monocyte chemoattractant protein 1, are a class of cytokines that guide the migration of blood borne inflammatory cells, such as neutrophils and macrophages, towards the source of the chemokine. Consequently, they play important roles in cellular communication and inflammatory cell recruitment. Expression of chemokines such as MCP-1, macrophage inflammatory protein-1α (MIP-1α), and fractakline following focal ischemia is thought to have a deleterious effect by increasing leukocyte infiltration [42]. The level of a variety of chemokines has been found to increase in animal models of ischemia and their inhibition or deficiency has been associated with reduced injury [43-45]. Mice without the chemokine receptor CCR2 are protected against ischemia-reperfusion injury [46].

### Cellular adhesion molecules

There is increasing evidence that cellular adhesion molecules (CAMs) play an important role in the pathophysiology of acute ischemic stroke [21]. CAMs are upregulated in the first days after stroke by various cytokines and are responsible for the adhesion and migration of the leukocytes. Leukocytes roll on the endothelial surface and then adhere to the endothelial cells. The interaction between leukocytes and the vascular endothelium is mediated by three main groups of CAMs: the selectins, the immunoglobulin gene superfamily, and the integrins. Selectins, especially E- and P-selectins are upregulated and mediate leukocyte rolling and recruitment during the early stages of ischemia [47] Among the immunoglobulin family member, intercellular adhesions molecule-1 (ICAM-1) and vascular cell adhesion molecule-1 have been the most extensively investigated in cerebral ischemia. Within hours after stroke onset, ICAM-1 expression increases upon stimulation by cytokines [48].

Patients with acute ischemic stroke had higher soluble intercellular adhesion molecule-1 (sICAM-1) levels compared to patients without cardiovascular disease. Moreover, sICAM-1 levels were significantly higher in patients who died compared to those who survived [49]. High sICAM-1 levels on admission are associated with early death is ischemic middle-aged stroke patients suggesting a pathogenic role of inflammation in the evolution of ischemic stroke.

A number of animal studies have shown that after transient and permanent focal ischemia the upregulation of CAMs, especially ICAM-1, P- and E-selectin, preceded the invasion of neutrophils into brain. There is ample evidence from animal models of MCAO that expression of CAMs is associated with cerebral infarct size. Thus, genetic ablation of CAMs resulted in reduced infarct size, which could be mimicked by treatment with anti-CAM antibodies [50,51]. Inhibition of leukocyte activation and infiltration into the ischemic cerebral tissue has, therefore, been an important area of neuroprotection research. Thus far, anti-CAM treatment has not been successful in patients with acute ischemic stroke. However, further translational research into the therapeutic targeting of CAM is ongoing.

The spatiotemporal profile of CAMs is still largely unresolved, even though they are crucial for efficient anti-inflammatory therapies. More knowledge of the spatiotemporal profile of CAMs may lead the way to successful application and monitoring of promising anti-inflammatory treatment strategies after stroke.

### Matrix metalloproteinases

MMPs are a family of proteolytic enzymes that are responsible for remodeling the extracellular matrix and that can degrade all its constituents. Expression of MMPs in the adult brain is very low to undetectable, but many MMPs are upregulated in the brain in response to injury [52]. Neurons, astrocytes, microglia, and endothelial cells have all been shown to express MMPs after injury. Stroke is associated with a biphasic disruption of the blood brain barrier (BBB) leading to vasogenic edema and hemorrhage and experimental studies have shown that that BBB breakdown and hemorrhage results from the expression and activation of MMPs [53].

MMP-2 and MMP-9 have been implicated in cerebral ischemia. Elevated MMP-9 levels were found in brain tissue and in serum from patients with acute ischemic stroke and in animal models of stroke beginning at 12 h after permanent MCAO [54]. MMP-9 is normally absent and this is the major MMP associated with neuroinflammation. Early (day 1) MMP-9 inhibition reduced infarction of day 14. However, benefit was lost when the treatment was delayed until day 3 and stroke pathology was exacerbated when administration was delayed until day 7 [55]. These studies all suggest that MMP inhibition could have a beneficial effect on the outcome of stroke but the effect will depend on the timing of treatment in relation to the stage of brain injury [55].

### Regulatory T lymphocytes

Severe brain ischemia also perturbs innate and adaptive immune cells, resulting in systemic immunodepression that predisposes patients after stroke to life-threatening infections [56]. Postischemic alterations in the immune system might represent a useful immunomodulatory adaptation, preventing autoimmune reactions against CNS antigens after stroke.

Recently, regulatory T lymphocytes (T_reg_) were shown to play an important role in protecting cells in a mouse model for stroke [57]. Thymus-derived CD4^+^CD25^+^Foxp3 T_reg _cells play a key part in controlling immune responses under physiological conditions and in various systemic and CNS inflammatory diseases [58]. T_reg _are generated by dendritic or antigen-presenting cells expressing the immunosuppressive mediator indoleamine 2,3-dioxygenase, the first enzyme in the kynurenine pathway, that degrades and converts tryptophan to kynurenine [59]. Interferon-γ and TNF-α which are both present at high levels in the ischemic brain induce IDO in response to chronic immune activation, possibly in microglia [60].

A stroke in mice with no functioning T_reg _cells in their blood caused much greater damage to the brain and greater disabilities than in animals with functioning T_reg _cells. T_reg _cells protect cells by suppressing the harmful activation of the immune system and can thus also prevent autoimmune diseases from developing. IL-10 is a cytokine that is produced by the T_reg _cells and seems to play an important role during a stroke. Mice with no functioning T_reg _cells that were injected with IL-10 on the first day following a stroke had markedly less brain damage than mice that did not receive IL-10. On the other hand, the transfer of genetically modified T_reg _cells unable to produce IL-10 offered no protection [57]. T_reg _cells producing IL-10 induce IDO suggesting that IL-10 may act upstream by modulating the production of IDO.

**Figure 4:**
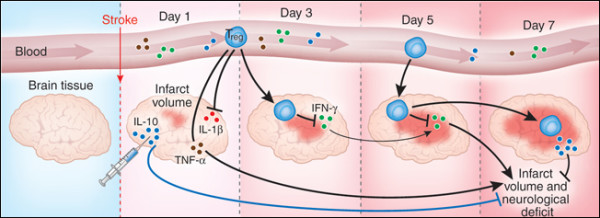
**Regulatory T (T_reg_) cells protect the brain after stroke**. Experiments by Liesz et al. [57] show that T_reg _cells prevent delayed lesion expansion in an IL-10-dependent manner in a mouse model of acute ischemic stroke. They also reduce the proinflammatory cytokine levels during the early postischemic inflammatory phase. Injection of IL-10 in the brain reduces infarct volume. Reprinted by permission from Macmillan Publishers Ltd: Nature Medicine 15, 138-139 Copyright 2009.

Depletion of T_reg _cells profoundly increased delayed brain damage and deteriorated functional outcome. Absence of T_reg _cells augmented postischemic activation of resident and invading inflammatory cells including microglia and T cells, the main sources of cerebral TNF-α and IFN-γ, respectively. T_reg _cells prevent secondary infarct growth by counteracting excessive production of proinflammatory cytokines and by modulating invasion and/or activation of lymphocytes and microglia in the ischemic brain. Liesz et al [57] found that T_reg _cells antagonize enhanced TNF-α and IFN-γ production, which induce delayed inflammatory brain damage, and that T_reg _cell-derived secretion of IL-10 is the key mediator of the cerebroprotective effect via suppression of proinflammatory cytokine production. IL-10 potently reduced infarct size in normal mice and prevented delayed lesion growth after T_reg _cells depletion (Figure 4).

### Post-stroke recovery

Patients experiencing a typical large-vessel acute ischemic stroke will lose 120 million neurons each hour. Compared with the normal rate of neuron loss during aging, the ischemic brain will age 3.6 years for every hour the stroke goes untreated. Thus, it is not surprising that the majority of stroke patients exhibit certain levels of motor weakness and sensory disturbances [2]. However, over time, most will show a certain degree of functional recovery which may be explained by brain reorganization and brain plasticity.

Brain plasticity refers to the brain's ability to change its structure and function during development, learning, and pathology. For example, within the minutes following ischemia, rapid changes are observed in the number and length of dendritic spines of the neurons in the penumbra region. The initial loss is then followed by the re-establishment of the dendritic spine synapses several months after the initial stroke as part of the functional recovery process [61].

Functional MRI studies have demonstrated that the damaged adult brain is able to reorganize to compensate for motor deficits [62,63]. The main mechanism underlying recovery of motor abilities appears to involve enhanced activity in preexisting networks. Studies in experimental stroke models demonstrate that focal cerebral ischemia promotes neurogenesis in the subventricular zone (SVZ) and subgranular zone (SGZ) of the dentate gyrus and induces SVZ neuroblast migration towards the ischemic boundary. More importantly, stroke-induced neurogenesis has also recently been demonstrated in the adult human brain, even in advanced age patients [64-66] These findings have led to a hope for a neurorestorative treatment of stroke which aims to manipulate endogenous neurogenesis and thereby enhance brain repair.

## Conclusion

In conclusion, in the presented work, we sought to provide a brief overview of the current understanding of inflammatory mechanisms involved during acute ischemic stroke and neuroprotective agents that can curtail neuroinflammation and could have utility in the treatment of stroke (see Table 1). As discussed, evidence suggests that post-ischemic oxidative stress and inflammation contribute to brain injury and to the expansion of the ischemic lesion. On the other hand, an adequate adaptive immune response after acute brain ischemia also plays an important role in response to ischemic injury as shown by the tremendous potential of T_reg _cells to prevent secondary infarct growth by counteracting the production of proinflammatory cytokines and by modulating the activation of lymphocytes and microglia in the ischemic brain [57]. These results provide new insights into the immunopathogenesis of acute ischemic stroke and could lead to new approaches that involve immune modulation using T_reg _cells.

To date, 1,026 drugs have been tested in various animal models, of which 114 underwent clinical evaluation [8]. The greater part of the agents studied until now have failed. Consequently, rt-PA remains the only agent shown to improve stroke outcome in clinical trials, despite the many clinical trials conducted. However, its use is limited by its short therapeutic window (three hours), by its complications derived essentially from the risk of hemorrhage, and by the potential damage by R/I injury. Because of these drawbacks the optimum treatment of cerebral focal ischemia remains one of the major challenges in clinical medicine.

## Abbreviations

ARE: Antioxidant response element; BDNF: brain-derived neutrotrophic factor; CAM: cell adhesion molecule; IGF-I: insulin-like growth factor I; IL: interleukin; IL-1R: interleukin-1 membrane receptor; IL-1RA: interleukin-1 receptor antagonist; Keap1: kelch-like erythroid cell-derived protein with CNC homology associated protein 1; MMP: matrix metalloproteinase; iNOS: nitric oxide synthase type II; ICAM-1: intracellular adhesion molecule 1; MCAO: middle cerebral artery occlusion; MCP-1: monocyte chemoattractant protein-1; NOS: nitric oxide synthase; Nrf2: nuclear factor erythroid-related factor 2; ROS: reactive oxygen species; rt-PA: recombinant tissue plasminogen activator; T_reg_: regulatory T lymphocytes; sICAM-1: soluble intracellular adhesion molecule 1; SOD: superoxide dismutase; t-BuOOH: tert-butylhydroperoxide; tBHQ: tert-butylhydroquinone; TGF: transforming growth factor; TNF-α: tumor necrosis factor-α.

## Competing interests

The authors declare that they have no competing interests.

## Authors' contributions

All authors participated in the preparation of the manuscript, and read and approved the final manuscript.
